# A Quasi-Experimental Study on the Effect of an Outdoor Physical Activity Program on the Well-Being of Older Chinese People in Hong Kong

**DOI:** 10.3390/ijerph19158950

**Published:** 2022-07-23

**Authors:** Daniel W. L. Lai, Xiaoting Ou, Jiahui Jin

**Affiliations:** 1 Faculty of Social Sciences, Hong Kong Baptist University, Hong Kong, China; jiahuijin@hkbu.edu.hk; 2 Department of Sport, Physical Education and Health, Hong Kong Baptist University, Hong Kong, China; 21481059@life.hkbu.edu.hk

**Keywords:** Chinese, older people, physical activity, happiness, mental health, quasi-experiment

## Abstract

Active participation in physical activity by older people is effective in improving their health. This research aims to examine the positive effects of participation in vigorous outdoor physical activities by older Chinese people in Hong Kong, and whether such effects would vary with socioeconomic background. A quasi-experimental, nonequivalent group design was used. A total of 22 participants were randomly assigned to participate in an outdoor physical activity program. Another 14 participants took part as a control group. The 14-item Self-Image of Aging Scale for Chinese Elders and the four-item self-report Subjective Happiness Scale were used to measure participants’ self-image and overall happiness level. All participants completed the assessment before and after the program. Happiness level was enhanced in participants in the experimental group (*p* = 0.037) and their level of overall mental health also improved (*p* = 0.031, η^2^ *p* = 0.129). Demographics did not have any significant effect on well-being outcomes. A structured outdoor physical activity program could be a viable choice for future practice to enhance the mental well-being of older Chinese people.

## 1. Introduction

For older people, engagement in moderate and vigorous physical activities, ranging from walking, gardening, and aerobic exercise, to fitness training [1,2], brings benefits to their physical, social, and emotional well-being. Older adults who exercise gain higher levels of balance, flexibility, and muscle strength compared with non-practitioners of exercise [3]. Engaging in physical activities is associated with improvement in physical fitness [3] and cognitive and executive functions [1]. A meta-analysis of randomized controlled trials has shown that low-frequency, long-term, regular aerobic exercise has benefited older people, whether they have intellectual problems or are undergoing physical rehabilitation [4]. In contrast, being inactive increases the risk of chronic disease among older people, who are generally more prone to multiple chronic conditions due to aging [2]. Thus, an exercise program should be considered a part of the care program for older people [5].

The benefits of physical activity programs also extend beyond physical health. Social well-being and psychological well-being are found to be associated with participation in physical activities [6]. Engaging in physical activities is found to be related to reduced risks of depression, anxiety, dementia, and Alzheimer’s disease [5,6]. It can contribute to enhanced quality of life and increased levels of self-concept and self-esteem [2,5]. Recent studies have also confirmed the positive impact of physical activity on happiness [7] and self-image [8].

However, it is not uncommon for older people to hesitate to engage in outdoor physical activities because some may consider these activities risky [9]. For instance, some older people may fear the risk of a heart attack, stroke, or even death, which hinders them from participating in strength and balance activities [9]. Furthermore, a lack of support from professionals or family may also discourage them from participating [10]. Additionally, cultural beliefs and values may be potential barriers to older people’s engagement in physical activities in different contexts. These beliefs can shape the different ways in which older people perceive risk and vulnerability and accordingly take action (or not) [11]. For instance, in Chinese society, passive leisure activities, which refer to less active activities, such as watching TV and listening to the radio, are generally considered preferable to intense physical activities [12]. In other words, it is important to examine the ways in which older Chinese adults view outdoor physical activities and evaluate their intensity, the impact of outdoor physical activities on their health, and perceived and actual challenges involved in participation. Additionally, there are differences in understanding how older adults perceive physical activities in different cultural contexts and how individual and group physical activities can generate different health outcomes [11]. Understanding this would help to address the beliefs and awareness associated with participation in physical activities (or lack of it) upheld by older adults, thereby informing the design of more culturally sensitive regular exercise programs for them.

To fill the research gap, this study aims to expand the existing literature by investigating older Chinese adults’ perceptions and experiences of participating in an outdoor physical group activity program as well as the impact of the program on their physical, mental, and social well-being. 

## 2. Methods

### 2.1. The Intervention

Outward Bound Hong Kong designed the outdoor physical activity program in collaboration with a lifelong learning program hosted by a local university in Hong Kong. The six-day program took place in December 2019 in the northeast part of Hong Kong near the Outward Bound Hong Kong Wong Wan Chau base. Project activities included visits to remote villages and locations on remote islands. The participants also took part in outdoor physical activities including sea kayaking, rafting, hiking, and overnight camping alone. All activities were conducted under the guidance of professionals. All participants underwent a health check prior to the program.

### 2.2. Research Design and Measures

A quasi-experimental non-equivalent group design was used. The experimental group consisted of the participants who took part in the Journey of Life—Active Ageing in the Outdoors program. The sample for the experimental group was comprised of some of the program participants. As this study focused on both aging and older adults, the program targeted the recruitment of people aged 55 and above in Hong Kong. The program was entitled “Journey of Life—Active Ageing in the Outdoors.” Open recruitment was done via social media platforms, websites, and information dissemination to members of the lifelong learning program. All participants had to go through health screening to ensure they were physically fit enough to take part in the program. Each participant was assigned a serial number and a lottery method was used to draw the numbers to be assigned to the intervention group and the control group. Due to the predetermined capacity of the program, 22 eligible participants were randomly selected to be included in the experimental group, while the remaining participants were treated as the control group. All participants were asked to complete the pre-test and post-test before the program began and also after the program ended. The participants in the experimental group would engage in outdoor activities under professional guidance, while those in the control group would receive no intervention. 

This study received ethics approval from the Human Subject Ethics Sub-Committee of The Hong Kong Polytechnic University (Approval Number: HSEARS20191125003). Written informed consent was obtained from all participants. All research activities were carried out in accordance with the relevant guidelines and regulations and in line with the Helsinki Declaration.

The key outcome variables included participants’ perception of self-image, self-rated mental health, and level of happiness. The 14-item Self-Image of Aging Scale for Chinese Elders was used to measure the participants’ perception of self-image rated on a scale of 1 to 5 and had an acceptable Cronbach’s alpha greater than 0.70 and a Guttman split-half reliability of 0.68 [13]. A higher score indicated a better self-image. Self-rated mental health was measured using a single item rated on a five-point scale of 1 (very poor) to 5 (very good). Overall happiness level was measured by the four-item self-reported Subjective Happiness Scale with each item rated on a seven-point scale, a higher score indicating a higher level of happiness, with a good internal consistency (α = 0.80~0.94) [14]. Examples of items include to what extent they identify themselves as being a happy person and their level of happiness relative to their peers.

Gender, age, marital status, education level, and financial status were the demographic variables measured. Financial status was rated on a five-point Likert scale ranging from 1 = very good to 5 = very poor.

### 2.3. Data Analysis

SPSS version 26.0 and R version 4.1.0 were used in data analysis. Descriptive statistics were used to examine the demographic and key outcome variables. t-test was adopted to evaluate the differences in the participants’ ages between the experimental and control groups. Fisher’s exact test was employed to evaluate the differences in participants’ gender, marital status, educational level, and financial status between the experimental and control groups, which was preferred to the chi-square test given the small sample size [15]. All dependent variables examined were tested for normality by the Shapiro–Wilk test [16], which is suggested for a small sample size (n < 20). Based on the results of the Shapiro–Wilk test, not all the dependent variables exhibited normal distribution in both experimental and control groups (i.e., self-image scores in pre-test). Thus, in this study, parametric tests (independent-sample t-test, paired-sample t-test, ANOVA, and ANCOVA) and non-parametric tests (Mann–Whitney U test, Wilcoxon signed-rank test, Kruskal–Wallis test, and Quade’s ANCOVA) were adopted for normally distributed and non-normally distributed variables respectively. Specifically, the independent-sample t-Test and the Mann–Whitney U test were employed to compare the baseline data and post-intervention data between experimental and control groups with the scores on the key outcome measures as the dependent variables and the experimental conditions as the independent variable. The paired-sample t-test and Wilcoxon signed-rank test were adopted for evaluating the changes within a group. Analysis of covariance (ANCOVA) was used to yield unbiased intervention estimates by assessing between-group differences including the pre-test scores as covariates. For non-normally distributed variable in both the experimental and control groups, i.e. self-rated mental health, Quade’s non-parametric analysis of covariance (Quade’s ANCOVA) was employed. Partial eta-squared (η2 p) was calculated to assess effect sizes where a value of 0.01 to 0.06 was considered small, 0.06 to 0.14 medium, and 0.14 or higher considered a large effect size.

Finally, considering the differences in demographic background between the experimental and control groups, and to assess how demographics would influence the effects of the intervention, data concerning gender, marital status, educational level, and financial status were also examined. The differences between groups of different demographics were assessed by comparing the pre-test scores. Independent-sample t-test or analysis of variance (ANOVA) was conducted with variables normally distributed in all groups. The Mann–Whitney U test or the Kruskal–Wallis test was conducted with variables not normally distributed in at least one group. Two-way analysis of covariance (ANCOVA) was further used to examine the effects of demographics on the post-test scores, with both the intervention status and demographic variables and their interaction term set as predicting variables, pre-test scores as covariates, and post-test scores as the dependent variables. For the variables showing non-normal distributions in at least one subgroup, such as self-image in the pre-test of the male experimental group, and happiness in the post-test of the married experimental group, two-way non-parametric Quade’s rank ANCOVA tests were performed. 

## 3. Results

### 3.1. Participants’ Demographics

Initially, 44 potential participants were recruited with 22 participants randomly selected for the experimental group while the remaining were kept in the control group. Only 14 participants in the control group completed the pre and post questionnaires. The missing cases were either unable to be contacted or did not show any interest in taking part in the post questionnaire, probably due to a lack of incentive to keep them engaged during the intervention period when they received no program of intervention or services. Participants’ demographic characteristics are shown in Table 1 and no significant differences were found between the two groups, except for gender (p = 0.018), with more male participants reported in the control group than in the experimental group. 

### 3.2. Differences between Experimental and Control Groups

Table 2 shows no significant differences in all variables between experimental and control groups in both the pre-test and post-test.

The *p* values shown in the last column were from Paired *t*-tests and Wilcoxon signed-rank tests, illustrating the pre-test vs. post-test change within the experimental or control group after intervention. The participants in the experimental group reported significant improvement in happiness (*p* = 0.037), while no change was found in the control group.

Table 3 shows the results of (Quade’s) ANCOVA with the pre-test scores as covariates. Compared with the control group, the experimental group reported significantly better mental health after the intervention (*p* = 0.031, η^2^ *p* = 0.129) but no change in self-image and happiness (*p* > 0.05).

### 3.3. Comparison among Demographic Groups

Financial status (good or below good) had a significant impact on self-image in baseline, participants in a good financial status had significantly higher levels of self-image (*p* = 0.009 < 0.05; M _good status_ = 58.9 (6.57) > M _below good status_ = 53.0 (5.94)). There were no other differences among demographic groups found in the pre-test.

The results of comparison of the post-test results within the grouped sub-datasets showed that the experimental group had significantly better mental health than the control group after the intervention for in females (*p* = 0.039 < 0.05; M _experimental_ = 4.24 (0.664) > M _control_ = 3.40 (0.547)) and in unmarried participants (*p* = 0.0495 < 0.05; M _experimental_ = 4.33 (0.707) > M _control_ = 3.50 (0.548)).

### 3.4. Effects of the Demographics on Intervention’s Outcome

Based on the two-way (Quade’s) ANCOVA which included intervention and gender, marital status, educational level, and financial status as covariates, a significant main effect of the intervention on mental health (*p* = 0.030~0.034) was found with medium effect size (η^2^ *p* = 0.093~0.138) and no interaction effects with the demographics were found. The results of two-way (Quade’s) ANCOVA are comparable to one-way (Quade’s) ANCOVA (*p* = 0.031, η^2^ *p* = 0.129).

## 4. Discussion

The key finding was that participation in outdoor activities was effective in improving older people’s happiness and mental health, although no statistically significant effects on older people’s self-image were found. The results support the positive impacts of group outdoor activity participation on older adults in Hong Kong.

Though the literature suggested the importance of older adults’ self-image [17], the self-image of the participants did not change in this study. Borglin, Edberg, and Hallberg [18] found that the self-image of older adults can be improved. Some studies on adolescents suggested that participation in outdoor activities could help improve self-image, but the interventions in these studies were conducted over 22 weeks [19]. Thus, the insufficient intensity of the current intervention may be a reason for the insignificant result.

There was a significant improvement in happiness among participants in the experimental group after the intervention. However, based on the results of ANCOVA, the effect of the intervention on happiness was no longer significant. This is probably because people’s levels of happiness are relatively stable, and the literature supports that an individual’s happiness is 50% or even 80% heritable and will not change significantly [20]. Therefore, outdoor activities can still be considered meaningful for enhancing levels of happiness. This finding is consistent with previous studies which found that exposure to the natural environment and outdoor activities participation could improve older people’s mental well-being and happiness [21]. Outdoor activities enhanced the happiness of older people and prevented them from spending most of their time at home and alone [22]. 

Based on the results of ANCOVA, mental health was enhanced after the intervention. In all models, the intervention showed significant main effects with moderate effect sizes. There are some studies which have reported the positive effects of outdoor or physical activity on mental health in the past [6,23]. Firdaus [24] suggested that outdoor activities provided a chance to access the natural environment which contributed to better mental health. Moreover, older people’s participation in different activities is important to maintain their social networks and stay socially active [25]. Similarly, Chang, Wray, and Lin [26] also clarified that mental health was found to be more affected by leisure activities than physical health.

## 5. Limitations

Despite the use of experimental and control groups in the design, due to the small sample size, we cannot exclude the possibility of type II error [27]. The small sample size also resulted in non-normal data, leading to the use of non-parametric tests. Moreover, the differences in demographic background between the experimental and control groups could be a concern, though efforts were made in this study to take into consideration the effects of demographic factors on the outcome variables. In future studies, larger samples with targeted demographic groups should be examined to clarify the potentially different effects of the intervention on aging adults with specific demographic backgrounds.

Another limitation is the low intensity of the intervention which could be the reason for the insignificant results and some other significant effects of outdoor activities on older adults may not have shown. Previous studies have found that short-term changes in mood may occur shortly after activities, but whether participation in these activities is meaningful in the long term still requires further research [28] using a longitudinal research design.

## 6. Conclusions

Active participation in outdoor physical activities by older people contributes to the improvement of their mental health. In future practice, structured outdoor physical activity programs should be further encouraged as a viable option for the physical and mental well-being benefits of older Chinese people.

## Figures and Tables

**Table 1:** Participants’ demographic characteristics.

Variables	Experimental Group (*n* = 22)	Control Group (*n* = 14)	Sig.
Age, mean (SD)	60.05 (4.24)	59.79 (5.25)	0.871 ^a^
Gender, *n* (%)			
Male	5 (22.7%)	9 (64.3%)	0.018 ^b,^*
Female	17 (77.3%)	5 (35.7%)	
Marital status, *n* (%)			
Married	13 (59.1%)	8 (57.1%)	1.000 ^b^
Single/Divorced/Widowed	9 (40.9%)	6 (42.9%)	
Educational level			
Secondary and below	9 (40.9%)	5 (35.7%)	1.000 ^b^
Non-degree diplomas	5 (22.7%)	3 (21.4%)	
University and above	8 (36.4%)	6 (42.9%)	
Financial status			
Good	11 (50%)	3 (21.4%)	0.160 ^b^
Below good	11 (50%)	11 (78.6%)	

Note. ^a^: *t*-Test; ^b^: Fisher’s exact test. * *p* < 0.05.

**Table 2:** Comparison of pre- and post-test between experimental and control groups and the change after intervention for the two groups.

Variables	Pre-Test	Post-Test	Sig. (Pre vs. Post)
Self-image			
Experimental gp	56.59 (6.01)	57.18 (6.94)	0.514 ^c^
Control gp	53.19 (7.55)	53.29 (8.31)	0.953 ^c^
Sig. (Exp vs. Control)	0.144 ^a^	0.137 ^a^	
Happiness			
Experimental gp	21.55 (3.08)	22.68 (3.37)	0.037 ^c,^*
Control gp	19.21 (4.06)	20.07 (4.51)	0.336 ^c^
Sig. (Exp vs. Control)	0.059 ^a^	0.055 ^a^	
Mental health			
Experimental gp	4.09 (0.68)	4.32 (0.65)	0.132 ^d^
Control gp	4.07 (0.83)	3.71 (1.07)	0.206 ^d^
Sig. (Exp vs. Control)	1.000 ^b^	0.089 ^b^	

Note. Data are mean (SD). ^a^: Independent-sample *t*-Test; ^b^: Independent-sample Mann-Whitney U Test; ^c^: Paired-sample *t*-Test; ^d^: Wilcoxon signed-rank test. * *p* < 0.05.

**Table 3:** The effects of the intervention on measured outcomes.

Variables	F	(Quade’s) ANCOVA *p*	η^2^ *p*
Self-image	0.303	0.585	0.009
Happiness	0.588	0.449	0.017
Mental health ^a^	5.039	0.031 *	0.129

Note. (Quade’s) ANCOVA with post-test scores as dependent variables, experimental or control group as independent variables, and pre-test scores as covariates. ^a^: calculated by Quade’s ANCOVA. η^2^ *p*: partial eta squared. * *p* < 0.05.

## Data Availability

Supporting data and data analysis materials are available from the corresponding author (Daniel, Lai) upon request.
